# Lactate Dehydrogenase-A (LDH-A) Preserves Cancer Stemness and Recruitment of Tumor-Associated Macrophages to Promote Breast Cancer Progression

**DOI:** 10.3389/fonc.2021.654452

**Published:** 2021-06-10

**Authors:** Shengnan Wang, Lingyu Ma, Ziyuan Wang, Huiwen He, Huilin Chen, Zhaojun Duan, Yuyang Li, Qin Si, Tsung-Hsien Chuang, Chong Chen, Yunping Luo

**Affiliations:** ^1^ Department of Immunology, Institute of Basic Medical Sciences, Chinese Academy of Medical Sciences, School of Basic Medicine, Peking Union Medical College, Beijing, China; ^2^ Collaborative Innovation Center for Biotherapy, Institute of Basic Medical Sciences, Chinese Academy of Medical Sciences, School of Basic Medicine, Peking Union Medical College, Beijing, China; ^3^ Immunology Research Center, National Health Research Institutes, Zhunan, Taiwan

**Keywords:** metabolism, cancer stem cells, tumor-associated macrophages, lactate dehydrogenase A, E-cadherin, CCL2

## Abstract

Increasing evidence reveals that breast cancer stem cells (BCSCs) subtypes with distinct properties are regulated by their abnormal metabolic changes; however, the specific molecular mechanism and its relationship with tumor microenvironment (TME) are not clear. In this study, we explored the mechanism of lactate dehydrogenase A (LDHA), a crucial glycolytic enzyme, in maintaining cancer stemness and BCSCs plasticity, and promoting the interaction of BCSCs with tumor associated macrophages (TAMs). Firstly, the expression of LDHA in breast cancer tissues was much higher than that in adjacent tissues and correlated with the clinical progression and prognosis of breast cancer patients based on The Cancer Genome Atlas (TCGA) data set. Moreover, the orthotopic tumor growth and pulmonary metastasis were remarkable inhibited in mice inoculated with 4T1-shLdha cells. Secondly, the properties of cancer stemness were significantly suppressed in MDA-MB-231-shLDHA or A549-shLDHA cancer cells, including the decrease of ALDH^+^ cells proportion, the repression of sphere formation and cellular migration, and the reduction of stemness genes (SOX2, OCT4, and NANOG) expression. However, the proportion of ALDH^+^ cells (epithelial-like BCSCs, E-BCSCs) was increased and the proportion of CD44^+^ CD24^−^ cells (mesenchyme-like BCSCs, M-BCSCs) was decreased after LDHA silencing, suggesting a regulatory role of LDHA in E-BCSCs/M-BCSCs transformation in mouse breast cancer cells. Thirdly, the expression of epithelial marker E-cadherin, proved to interact with LDHA, was obviously increased in LDHA-silencing cancer cells. The recruitment of TAMs and the secretion of CCL2 were dramatically reduced after LDHA was knocked down *in vitro* and *in vivo*. Taken together, LDHA mediates a vicious cycle of mutual promotion between BCSCs plasticity and TAMs infiltration, which may provide an effective treatment strategy by targeting LDHA for breast cancer patients.

## Introduction

Breast cancer stem cells (BCSCs), a small population of tumor cells with self-renewal ability and differentiation potential, have been proposed as a driving force in breast cancer initiation and dissemination (1, 2). Substantial evidence indicates that BCSCs are not homogeneous but compose of stem cells with different phenotypes and functions, which is known as BCSCs heterogeneity (3, 4). At present, BCSCs are mainly divided into two subtypes: one is mesenchymal, quiescent type marked by CD44^+^/CD24^−^ (M-BCSCs); the other is epithelial, proliferative type marked by ALDH^+^ (E-BCSCs) (5). During tumor progression, BCSCs convert between quiescent mesenchymal-like and proliferative epithelial-like states to acquire specific phenotypes stimulated by their niche cells, ultimately cause tumor relapse and metastasis. Therefore, intensive study on BCSCs heterogeneity may provide a more precise strategy to targeting BCSCs for breast cancer therapy.

Cancer cells exhibit high glycolysis even in the presence of sufficient oxygen, which is known as Warburg effect (6). This abnormal metabolism promotes proliferation and survival of tumor cells with elevated glucose uptake and lactate production, and agents targeting glycolysis may offer a therapeutic opportunity in clinical practice (7–9). Interestingly, recent work has shown that distinct BCSCs states (M- and E-BCSCs) response markedly different to the antiglycolytic treatment (10). Lactate dehydrogenase A (LDHA), a class of 2-hydroxy acid oxidoreductase, mediates the conversion of lactate from pyruvate, NADH from NAD^+^ in the last step of glycolysis (11). Intensive studies documenting that elevated LDHA has been associated with the progression of aggressive cancers in a variety of tumor types and different responses to LDHA targeted therapy are shown in clinical settings (12–14). Although LDHA is indicated to have an essential role in survival and proliferation of tumor cells, relatively little is known about the mechanisms of it on regulating stemness and BCSCs heterogeneity.

Tumor-associated macrophages (TAMs) account for 30% to 50% of all infiltrating inflammatory cells in the tumor microenvironment of breast cancer, which have significant impact on the tumor occurrence, development, and metastasis (15–18). Stemness-related properties of BCSCs, for example self-renewal, drug-resistance and metastasis initiation are intensively supported by their suitable niche and dynamic interplay between BCSCs and TAMs is one of the most important manner (19). However, the potential molecular link between BCSCs metabolism and tumor immune evasion is not well established in breast cancer. In the present study, using orthotopic syngeneic immunocompetent mouse model, we investigate the fundamental role of LDHA, a metabolic enzyme, in BCSCs maintenance and TAMs recruitment. We demonstrate a fundamental role of LDHA in negative regulation of epithelial marker E-cadherin during E/M-BCSCs transition, and increased tumoral infiltration of CCL2-responding TAMs.

## Materials and Methods

### Cell Lines and Reagents

MDA-MB-231, 293T, 4T1, and RAW264.7 were obtained from ATCC. All cell lines were cultured according to guidelines from ATCC. The medium was supplemented with 10% FBS (ThermoFisher Scientific, Cat. No. 10099-141) and 100 U/ml penicillin-streptomycin (ThermoFisher Scientific, Cat. No. 15140122). Bone marrow–derived macrophage (BMDM) was extracted from femur of 6- to 8-week-old female Balb/c mice and cultured with RPMI 1640 (Biological Industries, Cat. No. 01-100-1A, containing 30 ng/ml M-CSF (Peprotech, 315-02) for 6 days.

### Animal Experiments

The animal study was reviewed and approved by the Ethics Review Committee of Peking Union Medical College. Six- to 8-week-old female Balb/c mice were provided from the Institute of Basic Medical Science of Peking Union Medical College. 10^5^ 4T1 cells resuspended in 100-μl PBS were injected into the fourth mammary fat pad of each mouse and treated with oxamate or vehicle after tumor formation. The tumor size was measured and recorded on the 7th day after injection. On day 21 after injection, tumor, lung, and spleen in each mouse were harvested for tested by flow cytometry, hematoxylin, and eosin (H&E) staining and immunohistochemical (IHC) analyses.

### Western Blotting

The cells were lysed with protein extraction reagent (ThermoFisher Scientific, USA) and total protein was harvested after centrifuging at 14,000*g* for 10 min. The concentration of protein was measured by NanoDrop (ThermoFisher Scientific, USA). After being separated in Bis-Tris Gels (180-8008H, Tanon), total protein was transferred to 0.22-μm PVDF membranes (1620177, Bio-Rad), which were then incubated in 5% bovine serum albumin (BSA) for 1 h. Primary antibodies against LDHA (2012, CST), OCT4 (2840, CST) were used to incubate the membranes at 4°C overnight. After washing, the membranes were incubated with secondary antibodies (goat anti-rabbit IgG-HRP; 40295, Bioss) for 1 h and then detected using high-sig ECL Western Blotting Substrate (180-5001, Tanon). The bandings were quantified by ImageJ software.

### RNA Experiments

Total RNA was extracted by using Trizol reagent (ThermoFisher Scientific, USA) and 1 μg of total RNA was used for the synthesis of first-strand cDNA with HiScript II Q RT SuperMix for qPCR (+gDNA wiper) reagent (R223-01, Vazyme). Real-time PCR was performed with ChamQ SYBR qPCR Master Mix Reagent (Q311-02, Vazyme) and detected by CFX96 Real-Time PCR Detection System (Bio-Rad).

### Generation of Stable Cancer Cell Lines

Mouse shNC and shLdha PLKO.1 plasmid were designed and produced by Beijing Yimeiang Biotechnology Co. Ltd (sequence shLdha 1, 5′-CCGGGUUCCCAGUUAAGUCGUAUAACUCGAGUUAUACGACUUAACUGGGAACUUUUUG-3′; sequence shLdha 2, 5′-CCGGCGUCUCCCUGAAGUCUCUUAACUCGAGUUAAGAGACUUCAGGGAGACGUUUUUG-3′; sequence shLdha 3, 5′-CCGGCGUGAACAUCUUCAAGUUCAUCUCGAGAUGAACUUGAAGAUGUUCACGUUUUUG-3′). The plasmids were amplified by transformed into E. coli and then extracted with TIANprep Mini Plasmid Kit (DP106, TIANGEN). Objective plasmids and packaging plasmids were co-transfected into 293T cells by Lipofectamine™ 2000 Transfection Reagent (ThermoFisher Scientific, USA) and lentiviruses produced by 293T cells were collected. After infection with lentiviruses and 1-week puromycin treatment, stable cancer cell lines were generated successfully.

### Cell Migration Assay

2 × 10^6^ cells/ml tumor cells resuspended in 100 μl RPMI1640 (containing 1% FBS) were seeded into 8-μm pore polycarbonate filters, and complete medium (containing 10% FBS) was added into the bottom well. After 16 h culture, the non-migratory cells on the top of the filters were removed by cotton swab, and the migratory cells on the bottom of filters were dyed with 0.1% crystal violet for 10 min. The crystal violet could be eluted by 33% acetic acid, and the absorbance value of eluent was read out by Elisa (Biotec) at the wavelength of 570 nm.

### Flow Cytometry

The ALDH^+^ cells were detected by using ALDEFLUOR assay kit (01700, Stem Cell). 5 × 10^5^ cells were suspended in ALDH assay buffer containing substrate and then incubated at 37°C for 40 min with or without diethylaminobenzaldehyde (DEAB) reagent. The CD44^+^CD24^−^ CSCs were detected by using CD44-PE and CD24-APC antibodies incubated with tumor cells for 30 min at 4°C from light. Macrophages in tumor tissues were marked by CD45^+^F4/80^+^CD11b^+^ after single cell preparation. Suspensions of single cells were detected by flow cytometer (Millipore, Guava 5HT), and the data were analyzed by FlowJo software.

### Tumorsphere Formation Assay

Tumor cells were suspended in Mammocult medium (Stem Cell, Canada) at appropriate concentration and added into ultra-low plates (Corning, USA). The tumorspheres (sphere diameter ≧70 μm) were counted after 5 to 7 days of culture.

### ELISA

Culture supernatants of tumor cells was collected after centrifugation, and the concentration of CCL2 was measured by Mouse Uncoated ELISA Kit (88-7391-88, ThermoFisher Scientific, USA) according to providing protocol.

### Bioinformatics Analysis

From the GDC of TCGA, the clinical information and LDHA sequencing data of breast cancer patients were downloaded and sorted into a matrix. Patients were divided into tumor and paratumor groups according to source of tissue and compared in pair. Furthermore, patients were divided into three groups according to the expression of LDHA and then classified into four groups characterized by cancer clinical stage. In addition, patients were divided into negative and positive groups according to the distant metastasis information. With the statistical analysis, the correlation between LDHA expression and clinical stage or metastasis were obtained.

### Statistical Analysis

All results were presented as mean ± SD. Correlation between LDHA and clinical stage and metastasis of breast cancer in TCGA database was analyzed by one-way ANOVA test. Other results were analyzed with the Student’s t-test (unpaired). The data normality was established before the application of the statistical analysis. *P* < 0.05 was used as the criterion for statistical significance (*, *P* < 0.05; **, *P* < 0.01; ***, *P* < 0.001).

## Results

### LDHA Positively Correlated With Clinical Stages and Cancer Metastasis, and Might Be Involved in the Regulation of Tumor Immune Microenvironment

**Figure 1:**
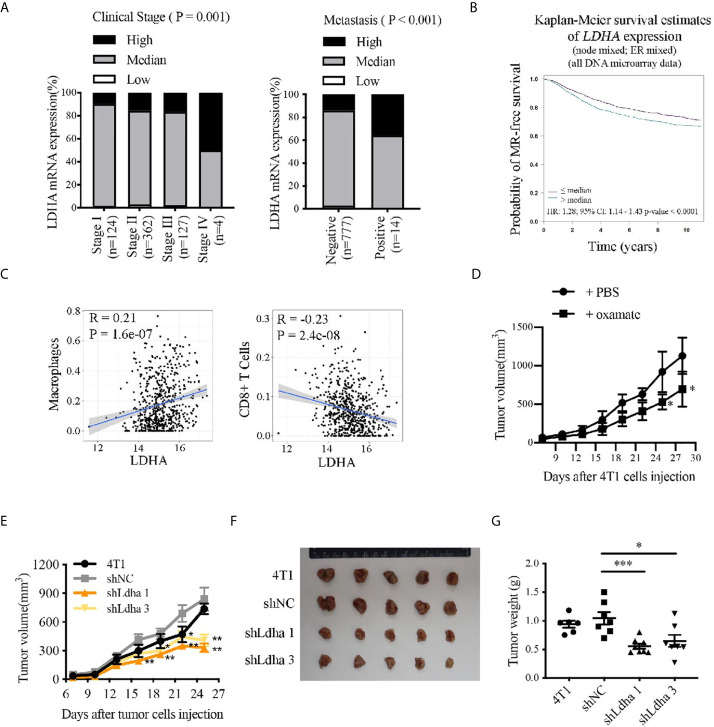
LDHA predicted progression and prognosis of breast cancer patients, correlated with immunocytes infiltration in TME and promoted 4T1 tumor growth in mouse. **(A)** Proportion of patients depend on the LDHA expression in each clinical stage. Breast cancer samples were classified as high, medium, or low LDHA expression according to log2 (FPKM of LDHA). **(B)** Kaplan-Meier analysis of the overall survival of breast cancer patients with LDHA high or low expression. The analysis was performed by Breast Cancer Gene-Expression Miner v4.4 in Integrated Center for Oncology. **(C)** Bioinformatics analysis of the correlation between LDHA expression and infiltration of macrophages or CD8 T cells in breast cancer based on TCGA data set. **(D)** Tumor growth curve of mice inoculated with 4T1 cells treated with PBS (n = 4) or oxamate (n = 5). **(E)** Tumor growth curve of mice inoculated with 4T1 wild type, shNC or shLdha cells (n = 8 in each group). 1×10^5^ cells were injected per mice. **(F)** Tumors were harvested on 25^th^ day after tumor cells inoculation and tumor picture was shown (n = 5). **(G)** Tumors weight were measured following tumor harvest (n = 8 in each group initially). *P < 0.05; **P < 0.01; ***P < 0.001.

To investigate the role of LDHA in breast cancer progression, we analyzed the correlation of LDHA expression with the tumor clinical stages, metastasis, and survival according to TCGA database. The results showed that LDHA expression was markedly increased in patients at tumor advanced-stage or in metastatic tumors (**Figure 1A**), and high expression of LDHA was significantly associated with short overall survival of breast cancer patients (**Figure 1B**, *P* < 0.0001). In addition, with TCGA data set, we analyzed the relationship between LDHA expression and macrophages/CD8^+^ T lymphocytes infiltration in tumor microenvironment for the first time. It was meaningful to find that LDHA expression was positively correlated with macrophages infiltration but negatively related with that in CD8^+^ T lymphocytes in breast cancer (**Figure 1C**). Abovementioned results suggested that LDHA played an important role in the development of human breast cancer, as well as the immunoregulation of tumor microenvironment.

### LDHA Was Essential for the Growth of Murine Breast Cancer in an Orthotopic Syngeneic Immunocompetent Mouse Model

Oxamate, a specific LDHA inhibitor, which could suppress LDHA activity (**Supplementary Figure 1A**) was used to verify the effect of LDHA on tumor generation and progression in mice. For the treatment strategy, oxamate, dissolved in PBS, was intraperitoneally injected into the Balb/c mice xenografted with 4T1 tumor every day for 3 weeks (**Supplementary Figure 1B**). During the treatment, tumor size was measured every 3 days and as expected, tumor growth was obviously restricted in mice treated with oxamate (**Figure 1D**). It is important to note that by using two stable 4T1 cell lines with Ldha knock-down (4T1-shLdha1 and 4T1-shLdha3), we found that tumor grew significantly more slowly in both 4T1-shLdha groups compared with the control mice after tumor cells inoculated in the 4th breast fat pad of Balb/c mice, and tumor weight at 25th day was much lower as well (**Figures 1E–G**). Taken together, growth of 4T1 tumors was significantly suppressed in mice by using LDHA-specific inhibitor or shRNA, which indicated an important role of LDHA in mouse breast cancer progression.

### LDHA Maintained Stemness-Related Properties of Breast Cancer Stem Cells

**Figure 2:**
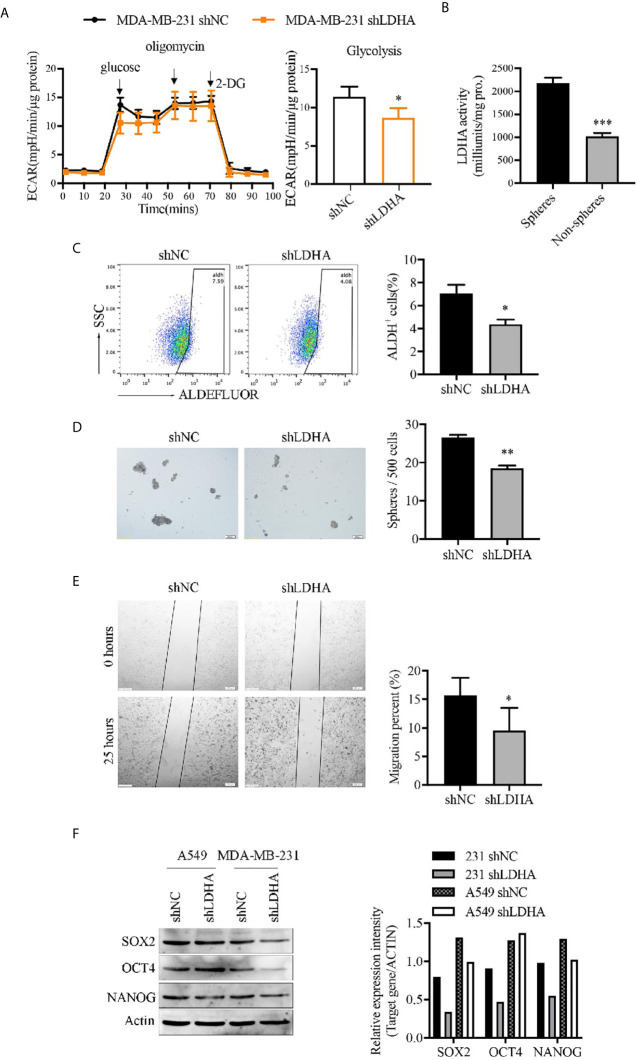
LDHA maintained stemness properties of human breast cancer cells. **(A)** Extracellular acidification rate (ECAR) of MDA-MB-231-shLDHA or control cells was detected by Seahorse Cellular Bioenergetics Analyzer. **(B)** LDH activity was detected in spheres or non-spheres cancer cells of MDA-MB-231 by colorimetry. **(C)** Percentage of ALDH^+^ cells in MDA-MB-231-shLDHA or control cells was analyzed by flow cytometry. **(D)** MDA-MB-231-shLDHA or control cells was cultured in sphere formation medium for 5-7 days, and the numbers of tumor-spheres were counted under a microscope. The representative images of spheres were shown on the left. Scale bar, 200 μm (insets). **(E)** Ability of cellular migration was measured by wound healing assay in MDA-MB-231 shNC and shLDHA cells. **(F)** Western blot analysis for SOX2, OCT4 and NANOG in A549-shLDHA, MDA-MB-231-shLDHA and their control cells. *P < 0.05; **P < 0.01; ***P < 0.001.

In consideration of elevated glycolysis in BCSCs from our previous study (20), and the important role of LDHA in maintaining glycolysis (**Figure 2A**), we presumed that LDHA was critical for keeping functionality of BCSCs. To demonstrate the role of LDHA in stemness maintenance, the expression of LDHA was detected in different breast cancer cell lines, and results have shown cells with higher degree of malignancy (HER2^+^ or basal−like type) have relative higher LDHA level (**Supplementary Figures 2A, B**). Tumor spheres of MDA-MB-231 cells cultured *in vitro* was enriched of CSCs population that was determined to have higher LDH activity compared with their adherent cells (**Figure 2B**). Next, stable cancer cell lines of MDA-MB-231-shLDHA, A549-shLDHA, and 4T1-shLdha were established, and both protein level and enzyme activity of LDHA/Ldha were significantly attenuated in LDHA knockdown cells (**Supplementary Figure 3**). Thereafter, the tumor spheres’ number, ALDH^+^ cell proportion, stemness genes (SOX2, OCT4, and NANOG), expression, and migratory cells were all reduced in MDA-MB-231-shLDHA cells (**Figures 2C–F**). Similarly, the expression of OCT4 also declined in 4T1-shLdha cells (**Supplementary Figure 4**). Both cellular proliferation and movement were suppressed but cellular apoptosis was rarely changed in 4T1-shLdha cancer cells (**Supplementary Figures 5A–C**). Taken together, our results highlighted a crucial role of LDHA in cancer stemness maintenance.

### LDHA Promoted the Transformation of M-BCSCs From E-BCSCs in 4T1 Cells

**Figure 3:**
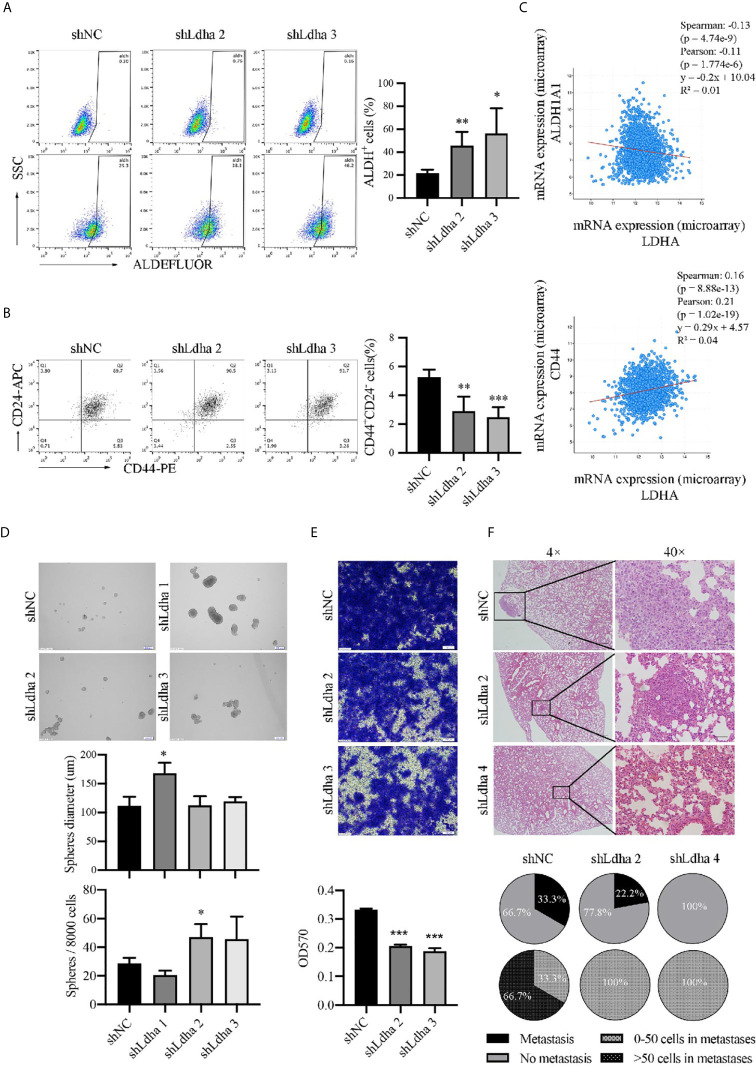
LDHA promoted E to M transition of BCSCs in mouse 4T1 cancer cells. **(A)** Percentage of ALDH^+^ cells in 4T1- shNC or 4T1-shLdha cells was analyzed by flow cytometry. **(B)** Percentage of CD44^+^CD24^−^ cells in 4T1- shNC or 4T1-shLdha cells was analyzed by flow cytometry. **(C)** Co-expression analysis of LDHA, ALDH1A1 and CD44 by using cBioportal for cancer genomics. **(D)** 4T1-shLDHA or control cells was cultured in sphere formation medium for 5-7 days, and the diameters and numbers of tumor-spheres were measured under a microscope. Scale bar, 200 μm (insets). **(E)** Cellular migration of 4T1-shNC or shLdha cells was measured by Transwell assay. Crystal violet was eluted by 33% acetic acid and determined at 570nm. **(F)** 4T1 shNC or shLdha cells was injected into 4^th^ breast fat pad of mice and lung was harvested on day 19 after injection. Tumor metastasis was analyzed by hematoxylin-eosin staining. Scale bar, 50 μm (insets). Number of mice with lung metastasis was 3/9(4T1 shNC), 2/9(shLdha 2) and 0/7(shLdha 4). The number of mice with more than 50 tumor cells in metastatic foci was 2/3(4T1 shNC), 0/2(shLdha 2) and 0/0(shLdha 4). *P < 0.05; **P < 0.01; ***P < 0.001.

ALDH^+^ cells represented a population of proliferative epithelial-BCSCs (E-BCSCs) and CD44^+^CD24^−^ cells enriched the metastatic mesenchymal-BCSCs (M-BCSCs) (5). It is interesting that the proportion of ALDH^+^ cells appeared to be increased and CD44^+^CD24^−^ population was reduced while LDHA was downregulated in 4T1 cells (**Figures 3A, B**), which suggested a regulatory effect of LDHA in the conversion between E-BCSCs and M-BCSCs. Furthermore, the co-expression analysis showed that LDHA was negatively correlated with the expression of ALDH1A1 but positively correlated with the expression of CD44 in human breast cancer samples based on the TCGA data set (**Figure 3C**). In addition, the formation of tumor spheres (enriched for proliferative BCSCs) was enhanced, but the migratory cells (enriched for metastatic BCSCs) were markedly reduced after silencing of LDHA in 4T1 cells (**Figures 3D, E**). It is important to note that pulmonary metastasis was also strongly suppressed in mice inoculated with 4T1-shLdha cells (**Figure 3F**). It was suggested that LDHA could preserve the proportion and properties of M-BCSCs; therefore, it was supposed to be a gatekeeper in BCSCs’ plasticity.

### Epithelial Marker E-Cadherin Was Negatively Regulated by LDHA

**Figure 4:**
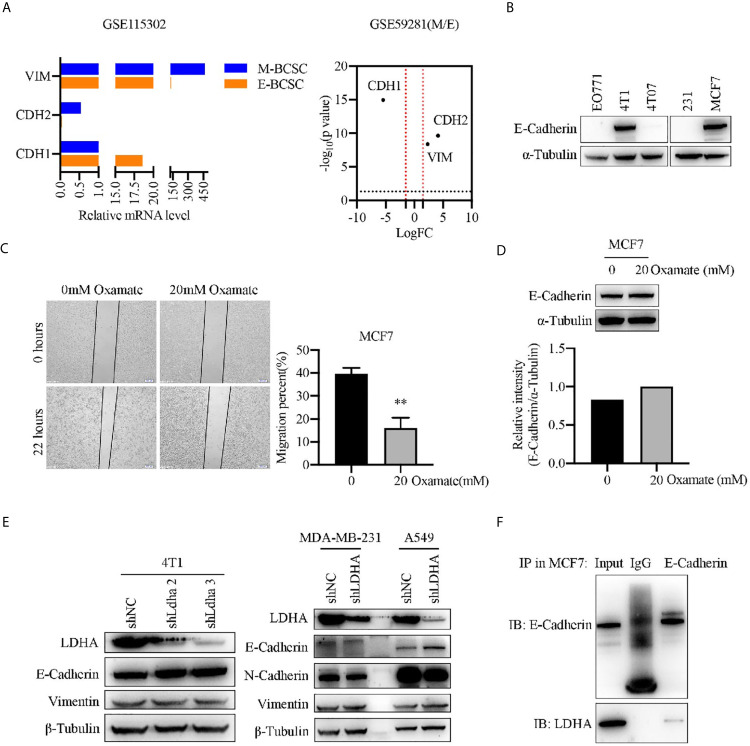
Epithelial marker E-cadherin was negatively regulated by LDHA. **(A)** Transcripts of CDH1, CDH2, and VIM were analyzed in E-BCSCs or M-BCSCs population based on GSE115302 and GSE59281 data sets. The horizontal dotted line represents *P* = 0.05, the vertical dotted line represents logFC = 2/−2. **(B)** Western blot analysis for E-Cadherin in EO771, 4T1, 4T07, MDA-MB-231 and MCF7 cells. **(C)** Cellular migration was measured by wound healing assay in MCF7 cells treated with 0 or 20 mM oxamate for 30 h. Scale bar, 200 μm (insets). **(D)** Western blot analysis for E-Cadherin in MCF7 cells treated with 0 or 20 mM oxamate for 30 h. **(E)** Western blot analysis for LDHA, E-Cadherin, N-Cadherin and Vimentin in A549-shLDHA or MDA-MB-231-shLDHA cancer cells. **(F)** Immunoprecipitation analysis of the interaction between LDHA and E-Cadherin in MCF7 cells. **P < 0.01.

To investigate the mechanism of LDHA regulating BCSCs heterogeneity, we performed a bioinformatics analysis by using GSE115302 and GSE59281 data sets. CDH1 encoding E-Cadherin was an epithelial marker, and CDH2/VIM encoding N-Cadherin/vimentin, respectively, represented the mesenchymal markers. As expected, CDH1 was highly expressed in E-BCSCs and CDH2/VIM were highly expressed in M-BCSCs (**Figure 4A**), which suggested that the EMT markers could be used to distinguish those two BCSCs subgroups. It was noteworthy that the migratory cells of MCF-7 treated with oxamate were reduced, and E-Cadherin level was increased correspondingly (**Figures 4C, D**). The expression of E-Cadherin which was overexpressed in 4T1 and MCF-7 cells (**Figure 4B**) was up-regulated upon LDHA knocked down (**Figure 4E**). Immunoprecipitation assay revealed that endogenous LDHA could interact with E-cadherin in MCF7 cells (**Figure 4F**). These results suggested that E-cadherin was negatively regulated by LDHA in M-BCSCs, which perhaps contributed the E/M-BCSCs transformation.

### LDHA Promoted the Tumoral Infiltration of TAMs in a 4T1 Murine Breast Cancer Model

**Figure 5:**
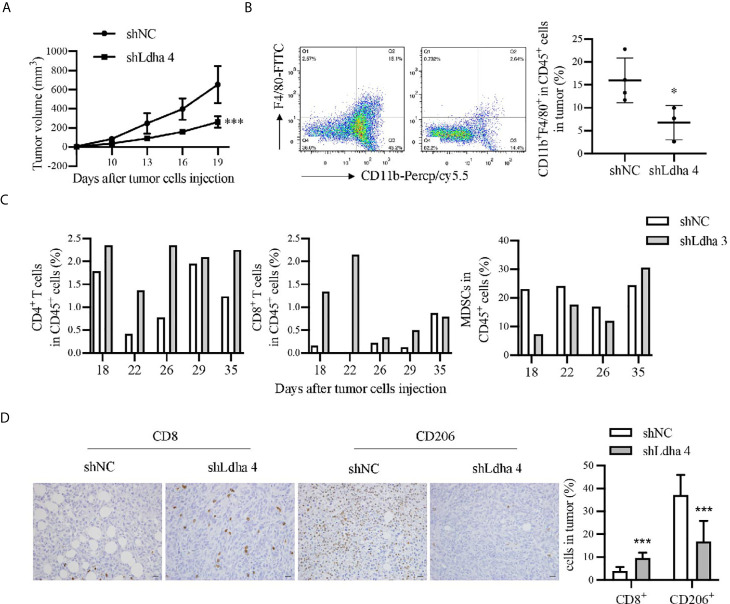
LDHA promoted tumoral infiltration of TAMs in 4T1 murine breast cancer model. **(A)** Tumor growth curve of mice inoculated with 4T1-shNC or 4T1-shLdha cells. 1×10^5^ cells per mice were injected into the fourth breast fat pad of Balb/c mice (n = 9 in 4T1-shNC group, n = 7 in 4T1 shLdha group). **(B)** Percentage of CD11b^+^F4/80^+^ macrophages in CD45^+^ immune cells were analyzed by flow cytometry. Tumor were harvested on day 19 after the tumor cells injection. CD45-PE, CD11b-Percp/cy5.5 and F4/80-FITC were used to mark macrophages in tumor. **(C)** Percentage of CD3^+^CD8^+^ T cells, CD3^+^CD4^+^ T cells or CD11b^+^Gr1^+^ MDSCs in CD45^+^ immune cells were analyzed by flow cytometry. Tumor were harvested on different days after the tumor cells injection (n = 1 in each group). CD45-PE, CD3ϵ-FITC, CD4-APC and CD8α-APC were used to mark T cells in tumor. CD11b-FITC and Gr1-APC were used to mark MDSCs in tumor. **(D)** The expression of CD8 and CD206 was analyzed by immunohistochemistry in 4T1 shNC or shLdha-injected tumors (n = 9 in 4T1-shNC group, n = 7 in 4T1 shLdha group). Scale bar, 20 μm (insets). *P < 0.05; ***P < 0.001.

**Figure 6:**
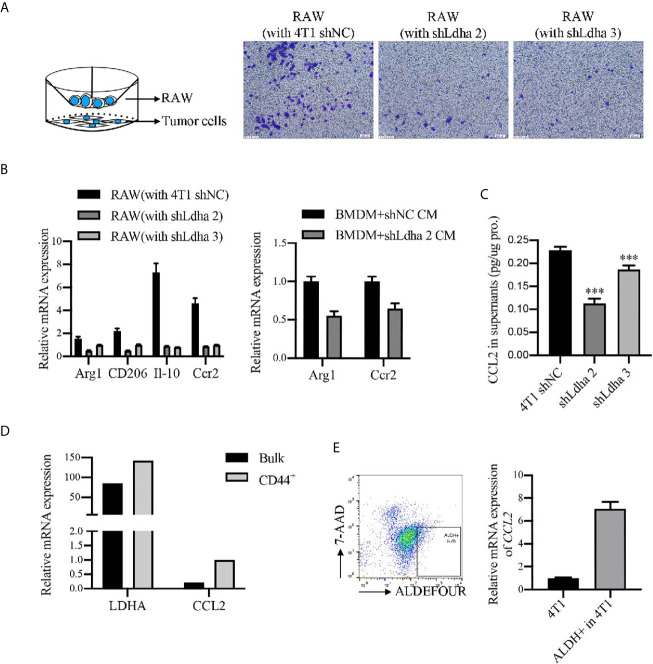
LDHA enhanced the recruitment and M2 phenotypes of macrophages *in vitro*. **(A)** Migratory capacity of RAW264.7 cells was analyzed by Transwell assay. RAW264.7 cells were seeded in 8-μm-pore polycarbonate filters and 4T1-shNC or 4T1-shLdha cells were seeded in the bottom well. Representative images were visualized by microscope (right) and percentage of migrating cells was calculated by use of ImageJ. Scale bar, 200 μm (insets). **(B)** Relative mRNA level of M2 markers in RAW 264.7 or BMDM cultured with conditional medium from 4T1-shNC or 4T1-shLdha cancer cells were detected by RT-qPCR. **(C)** Ccl2 level in the cultural supernatant from 4T1-shNC or 4T1-shLdha cells was detected by ELISA. Results were normalized by protein content in cells. **(D)** Relative mRNA level of LDHA and CCL2 in M-BCSCs or bulk cells were analyzed according to GSE115302 data set. **(E)** The mRNA level of Ccl2 was measured by RT-qPCR in ALDH^+^ or bulk 4T1 cells. ALDH^+^7-AAD^-^ cells were sorted out by flow cytometry. Error bars mean ± SD from three independent experiments. ***P < 0.001.

Previous studies demonstrated that tumor immune microenvironment, especially tumor-associated macrophages (TAMs) served as the key niche cell for CSCs maintenance (21–23). Using the 4T1 orthotopic breast tumor model in mice, besides a remarkable retardation of tumor growth (**Figure 5A**), the infiltration of macrophages, marked by CD45^+^CD11b^+^F4/80^+^, was significantly suppressed in 4T1-shLdha tumors (**Figure 5B**). Furthermore, the antineoplastic immune cells, including CD8^+^ or CD4^+^ T cells was highly increased, nevertheless the bone marrow-derived immunosuppressive cells (MDSCs) decreased sharply in 4T1-shLdha tumors (**Figure 5C**). It was also verified with immunohistochemistry that the numbers of CD8^+^ T cells was increased and CD206^+^ M2 macrophages was decreased infiltrated in 4T1-shLdha tumors (**Figure 5D**). Next, we explored the migratory ability and immunologic phenotype of macrophages cultured with diverse 4T1 conditional medium *in vitro*. It was notable that migratory RAW264.7 cells were dramatically decreased and the expression of M2 related genes (Arg1, CD206, IL-10, and Ccr2) in RAW264.7 or bone marrow derived macrophages (BMDMs) were markedly reduced when cultured with the conditional medium from 4T1-shLdha cells (**Figures 6A, B** and **Supplementary Figure 6**). Ccr2-expressing monocytes were effectively attracted into tumor sites by Ccl2 (24), and our results revealed that Ccl2 secreted from 4T1-shLdha cells notably decreased compared to the control cells detected by ELISA (**Figure 6C**). Intriguingly, bioinformatic analysis revealed that CCL2 and LDHA were both strongly increased in M-BCSCs population (CD44^+^ cancer cells) compared with the bulk cancer cells according to the GSE115302 data set (**Figure 6D**). Moreover, the expression of Ccl2 was significantly increased in sorted E-BCSCs (ALDH^+^ cancer cells) compared with the unsorted bulk 4T1 cells (**Figure 6E**). Taken together, these results suggested that tumoral infiltration of TAMs in breast cancer was intensively facilitated by Ldha/Ccl2 signaling pathway in BCSCs.

## Discussion

BCSCs are considered as key drivers of tumor growth, relapse, and metastasis, of which targeting the specific metabolism may help to eradicate this small subset of cells. The main metabolic pathway of cancer cell is aerobic glycolysis rather than oxidative phosphorylation, which is called “Warburg effect” and has been verified in many types of tumor (12, 13, 25–27). Our previous study revealed a higher glycolytic metabolism in BCSCs (20), but the mechanism of glycolysis maintaining stemness was not much clear. LDHA, a rate-limiting enzyme in glycolytic process, promotes tumor growth through variety of ways, which is verified to play an important role in stemness maintenance of BCSCs in this study. This result is in agreement with a recent report documenting that targeting LDHA inhibits lung tumor-initiating cells by using an inducible murine model (13). Recent evidence implies BCSCs are not a simplex cell population but mainly consist of two subgroups, which are termed as E-BCSCs and M-BCSCs. E-BCSCs are epithelioid, proliferative cancer stem cells, highly expressing epithelial-like marker E-Cadherin, while M-BCSCs are quiescent, metastatic cancer stem cells, highly expressing mesenchymal-like marker N-Cadherin. A combinatory approach targeting both M- and E-BCSCs illustrates a novel treatment approach targeting both BCSC states (28). For the first time, our results reveal that LDHA play a critical role in BCSCs heterogeneity, which indicated a special effect on E/M-BCSCs transition in 4T1 cells.

The transition of BCSCs from the E to the M state closely resembles the epithelial-to-mesenchymal transition (EMT), which plays an important role in the metastatic process of tumor cells (29). Our results also demonstrate a suppressive effect of LDHA on the epithelia marker E-Cadherin, which seemingly explains its essential function in E/M-BCSCs transition. Departure from cell membrane of E-Cadherin provoked by ubiquitylate degradation is an important mechanism for tumor cells to undergo EMT and enhance their metastatic ability (30, 31). In our data, ubiquitin of E-Cadherin was also detected in LDHA pull-down sample (data not shown). Therefore, we speculate that LDHA might promote the ubiquitination and endocytosis of E-Cadherin to facilitate the transformation of CSCs from E-BCSCs to M-BCSCs ultimately and it is also suggested that solely suppression of LDHA may not be sufficient to eliminate BCSCs containing different subpopulations. Fully investigation of metabolic heterogeneity in diverse subsets of BCSCs appears urgent and therapeutic strategies by targeting BCSCs’ diverse metabolism seems to have great prospect for breast cancer treatment.

The equilibrium of diverse BCSCs subgroups is regulated by the TME *via* multifaceted mechanisms including cytokine or chemokine signaling (5, 32, 33). Macrophages constitute up to 50% of the tumor infiltrating cells in human breast cancer and thus represent most non-neoplastic cells in the tumors. Our previous studies revealed that tumor associated macrophages promoted the stemness phenotypes of BCSCs by activating key inflammatory pathways (34, 35). In turn, this study demonstrated that LDHA, a cancer stemness promoter, could impede the infiltration of anti-tumor immune cells (CD4^+^/CD8^+^ T cells) and enhance the accumulation of the pro-tumoral immune cells (MDSCs and TAMs) in TME. Previous study revealed that the polarization of M2 phenotype was promoted by lactate in TAMs (36). Recent study revealed that lactate concentration quantified by the double quantum filtered (DQF) MRS, a non-invasive imaging method, is a sensitive marker for the prediction of tumor grades and prognosis in breast cancer. However, there has been study showing that silencing LDHA failed to alter lactic acid production in breast cancer cell line (37). Except for lactic acid, carbonic anhydrase 9 (CA-9), maintaining the acidic condition of TME, also contributed to the tumor progression (38). Interestingly, the lipid composition in TME is also an important factor for macrophages recruitment and functional remolding (39). In our study, by using the co-culturing system *in vitro*, we find that the recruitment of macrophages is strongly enhanced by LDHA. Mechanistically, this occurs at least in part through elevated secretion of macrophages-attracted chemokine CCL2 induced by LDHA. Recruitment of inflammatory monocytes/macrophages responding to CCL2 is critical for tumor cell pulmonary seeding (40).These findings revealed the different mechanisms of metabolic enzyme in regulating the TME and might provide novel targets for cancer therapy. Interestingly, CCL2 expression is dramatically increased in BCSCs based on TCGA data set and we propose a hypothesis that secretion of CCL2 from BCSCs is enhanced by LDHA, which could recruit more CCR2-positive tumor associated macrophages (TAMs) to further maintained the stemness of BCSCs. Moreover, E-Cadherin, the molecular marker of E-BCSCs, was suppressed by LDHA that partially explain the phenomenon of LDHA mediated E to M transformation in BCSCs. This hypothesis needs to be further fully investigated and targeting LDHA combined with CCL2/CCR2 antagonists may provide a better therapeutic outcome for breast cancer.

**Figure 7:**
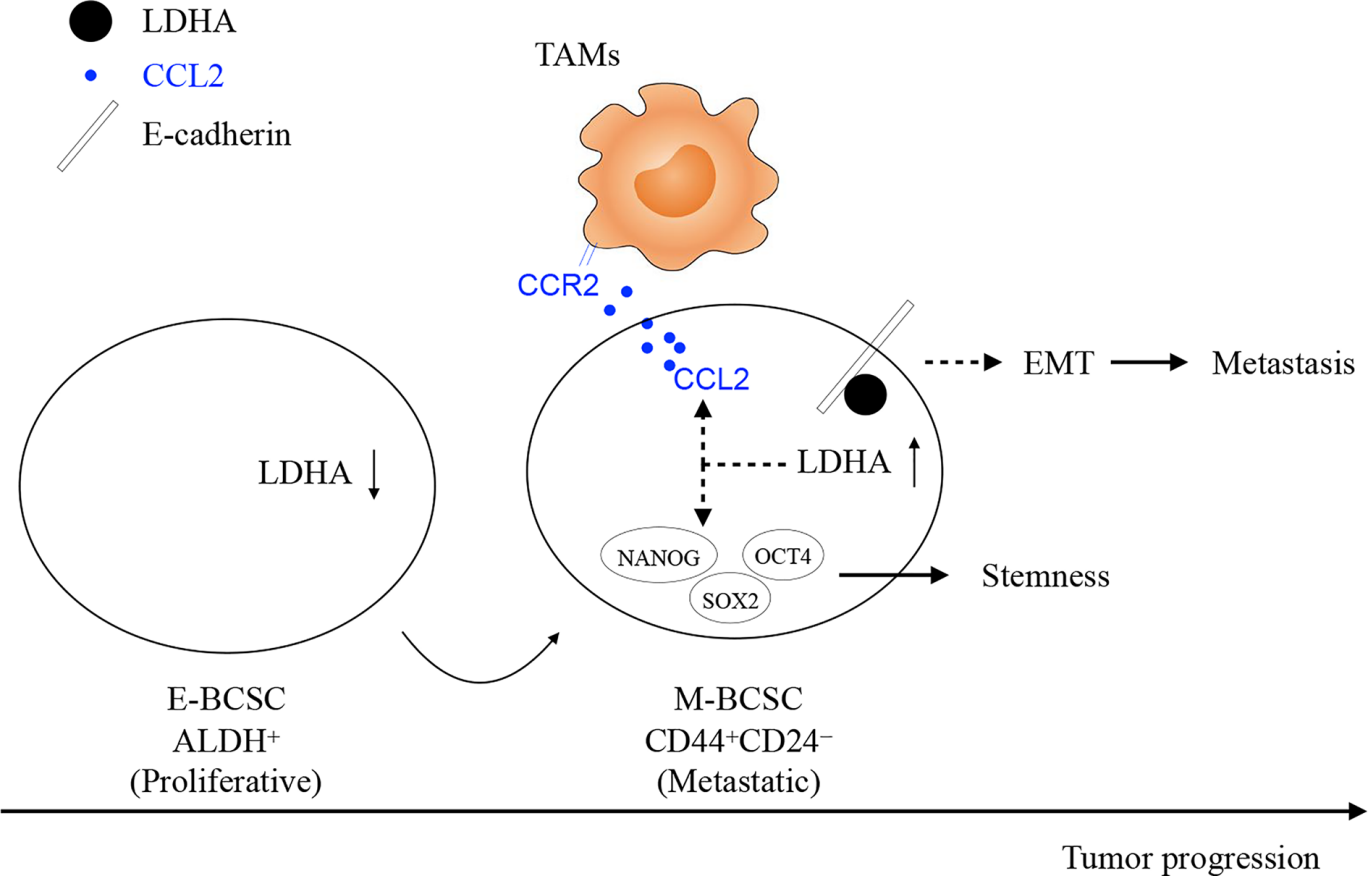
The work model of glycolytic enzyme of LDHA not only maintains the stemness and heterogeneity of BCSCs but also facilitates the infiltration of TAMs to trigger immune escape of BCSCs. At the early stage of breast cancer, E-BCSCs promote tumor rapidly growth and recruit more CCR2-expressing monocytes/macrophages and then, LDHA reduces E-Cadherin expression and increases CCL2 secretion in M-BCSCs to facilitated tumor metastasis at the advanced stage of breast cancer.

In conclusion, our study demonstrates that an essential glycolytic enzyme of LDHA not only maintains the stemness and heterogeneity of BCSCs but also facilitates the infiltration of TAMs to trigger immune escape of BCSCs (**Figure 7**). The mechanisms of tumor abnormal metabolism identified here can inform translational studies by targeting heterogeneous population and supportive niche of BCSCs, to develop more efficient treatment for breast cancer.

## Data Availability Statement

The original contributions presented in the study are included in the article/**Supplementary Material**. Further inquiries can be directed to the corresponding authors.

## Ethics Statement

The animal study was reviewed and approved by institutional review board of Institute of Basic Medical Sciences, Chinese Academy of Medical Sciences.

## Author Contributions

SW carried out the cellular and animal experiments. ZW carried out the bioinformatic analysis. SW and LM performed the statistical analysis. CC and SW drafted the manuscript. HH, HC, ZD, YLi, QS, and T-HC provided material support. CC and YLu conceived of the study and participated in its design. All authors contributed to the article and approved the submitted version.

## Funding

This work was supported by grants from the National Natural Science Foundation of China (NSFC): grant no. 81972795 and 81672914; the Bilateral Inter-Governmental S&T Cooperation Project from Ministry of Science and Technology of China: grant no. 2018YFE0114300; the National Basic Research Program (973) of China: grant no. 2013CB967202.

## Conflict of Interest

The authors declare that the research was conducted in the absence of any commercial or financial relationships that could be construed as a potential conflict of interest.
