# Supplementary material for: Lactate Dehydrogenase-A (LDH-A) Preserves Cancer Stemness and Recruitment of Tumor-Associated Macrophages to Promote Breast Cancer Progression
Source: Front Oncol. 2021 Jun 10;11:654452. doi: 10.3389/fonc.2021.654452 (PMC8225328; doi:10.3389/fonc.2021.654452)
Supplement: Supplementary file 1 [file DataSheet_1.pdf]

Supplementary Material

Supplementary Figures:

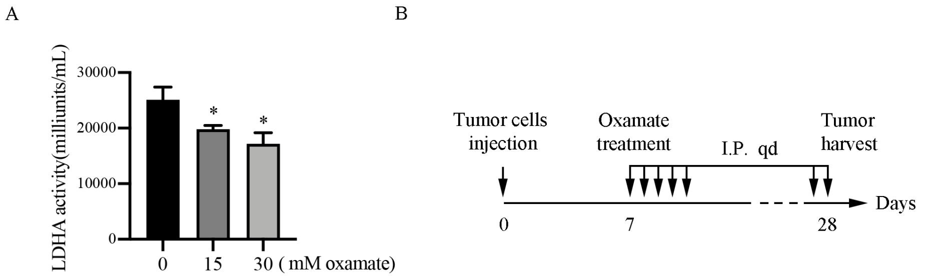

Supplementary Figure 1

4T1 cells or mice injected with 4T1 cells were treated with oxamate.

A) The activity of LDHA was suppressed by LDHA inhibitor oxamate in 4T1 cells. 4T1 cells were treated with PBS or oxamate dissolved in PBS (15mM, 30mM) for 30h and LDHA activity was detected by colorimetry. *, $P < 0.05$. B) Experimental scheme for application of oxamate treatment in mice.

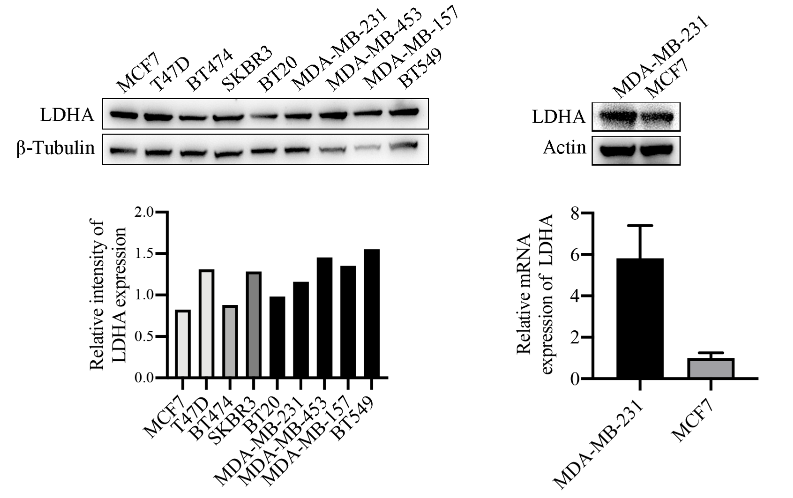

Supplementary Figure 2

The expression of LDHA in a variety of breast cancer cell lines was analyzed by using western blot and real-time qPCR.

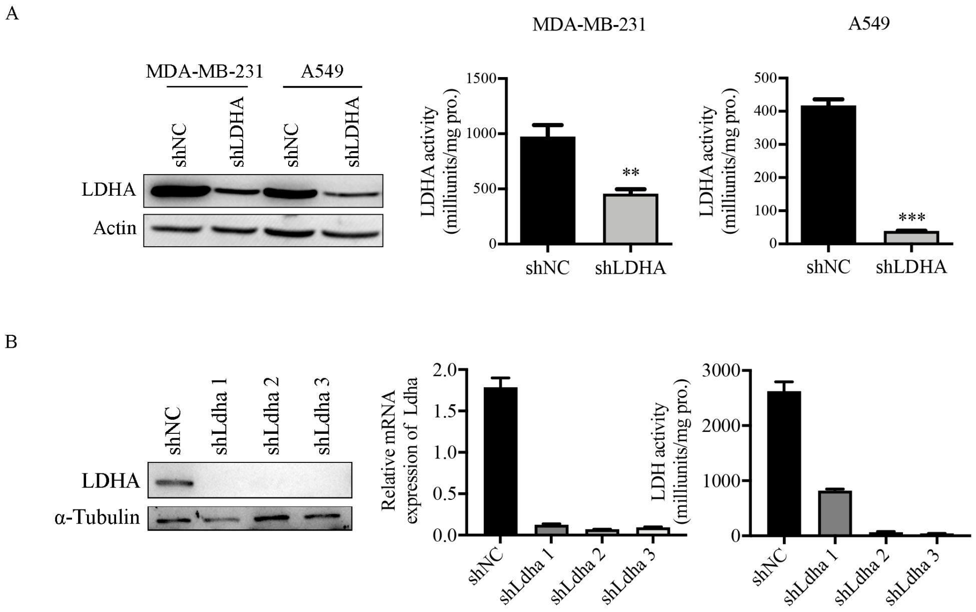

Supplementary Figure 3

Establishment and verification of LDHA-knockdown cancer cell lines.

A) LDHA expression was analyzed by western blot or qRT-PCR and LDH activity was detected by colorimetry in MDA-MB-231-shLDHA, A549-shLDHA or corresponding control cells. B) LDHA expression was analyzed by western blot or qRT-PCR and LDH activity was detected by colorimetry in 4T1-shLDHA or control cells. **, $P < 0.01$; ***, $P < 0.001$.

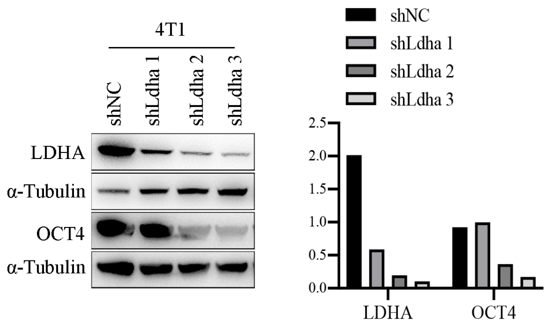

Supplementary Figure 4

LDHA and OCT4 expression in 4T1-shNC or 4T1-shLdha cells were analyzed by western blot.

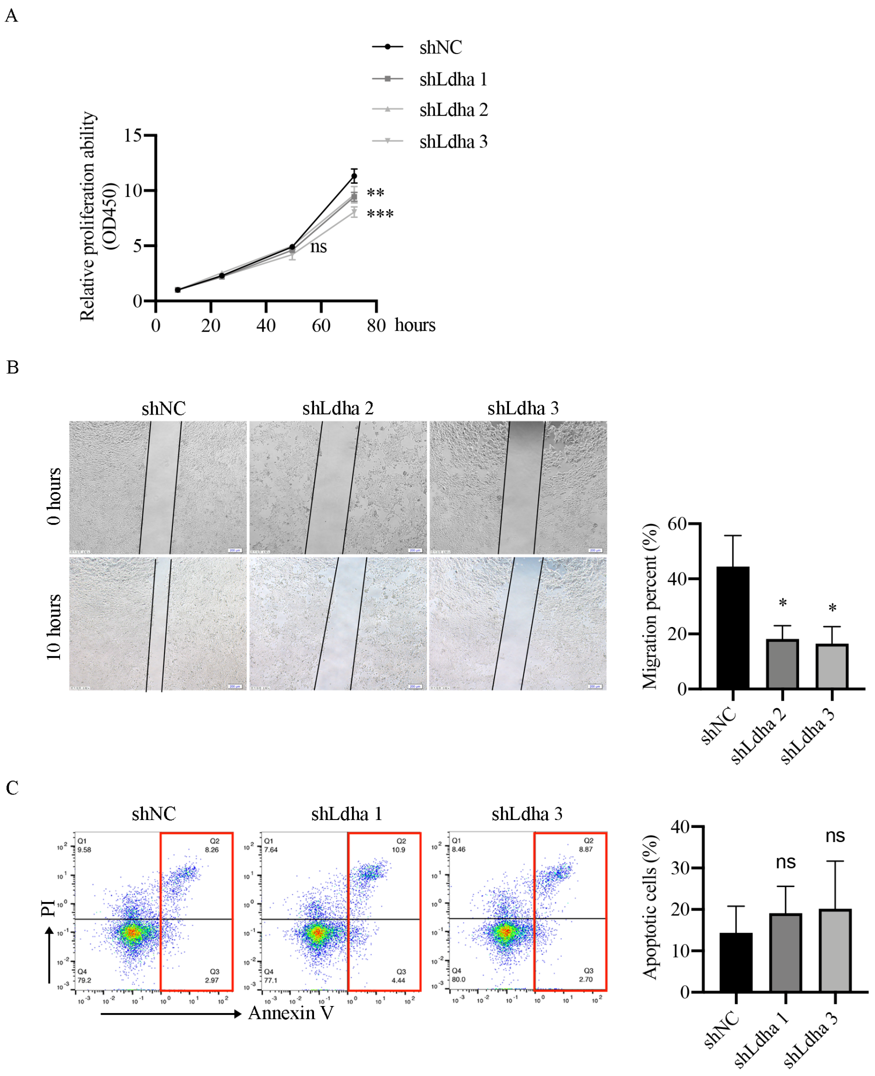

Supplementary Figure 5

The proliferation, movement, and apoptosis were analyzed in 4T1-shLdha cancer cells.

A) Proliferation of 4T1-shNC or 4T1-shLdha cells was measured by CCK-8 assay for 72 hours and OD450 was measured every 24 hours. B) Wound healing assay was

carried out in 4T1-shNC or 4T1-shLdha cells. C) Cellular apoptosis in 4T1-shNC or 4T1-shLdha cells was measured by flow cytometry. ns, no significance. *, $P < 0.05$; **, $P < 0.01$; ***, $P < 0.001$.

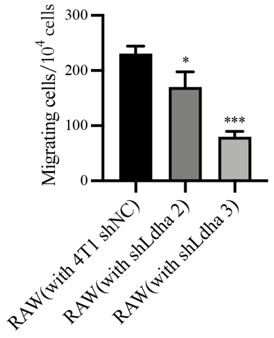

Supplementary Figure 6

Statistical analysis of migratory capacity of RAW264.7 cells co-cultured with 4T1 shNC or shLdha cells. Counts of Migrating cells were measured by use of ImageJ. *, $P < 0.05$; ***, $P < 0.001$.
